# The development of quick, robust, quantitative phenotypic assays for describing the host–nonhost landscape to stripe rust

**DOI:** 10.3389/fpls.2015.00876

**Published:** 2015-10-27

**Authors:** Andrew M. Dawson, Jan Bettgenhaeuser, Matthew Gardiner, Phon Green, Inmaculada Hernández-Pinzón, Amelia Hubbard, Matthew J. Moscou

**Affiliations:** ^1^The Sainsbury Laboratory, Norwich Research ParkNorwich, UK; ^2^National Institute of Agricultural BotanyCambridge, UK

**Keywords:** nonhost resistance, inappropriate pathogen, formae speciales, yellow rust, *Puccinia striiformis*, *Brachypodium distachyon*, barley

## Abstract

Nonhost resistance is often conceptualized as a qualitative separation from host resistance. Classification into these two states is generally facile, as they fail to fully describe the range of states that exist in the transition from host to nonhost. This poses a problem when studying pathosystems that cannot be classified as either host or nonhost due to their intermediate status relative to these two extremes. In this study, we investigate the efficacy of the Poaceae-stripe rust (*Puccinia striiformis* Westend.) interaction for describing the host–nonhost landscape. First, using barley (*Hordeum vulgare* L.) and *Brachypodium distachyon* (L.) P. Beauv. We observed that macroscopic symptoms of chlorosis and leaf browning were associated with hyphal colonization by *P. striiformis* f. sp. *tritici*, respectively. This prompted us to adapt a protocol for visualizing fungal structures into a phenotypic assay that estimates the percent of leaf colonized. Use of this assay in intermediate host and intermediate nonhost systems found the frequency of infection decreases with evolutionary divergence from the host species. Similarly, we observed that the pathogen’s ability to complete its life cycle decreased faster than its ability to colonize leaf tissue, with no incidence of pustules observed in the intermediate nonhost system and significantly reduced pustule formation in the intermediate host system as compared to the host system, barley-*P. striiformis* f. sp. *hordei*. By leveraging the stripe rust pathosystem in conjunction with macroscopic and microscopic phenotypic assays, we now hope to dissect the genetic architecture of intermediate host and intermediate nonhost resistance using structured populations in barley and *B. distachyon*.

## Introduction

Plants have a remarkable ability to resist the majority of pathogenic microbes they encounter. It is now widely posited that the molecular networks underlying this resistance are multi-factorial and can depend upon active or passive defense mechanisms (Thordal-Christensen, 2003; Fan and Doerner, 2012). While the individual contribution of each mechanism is hard to quantify, their common objective is to provide barriers that impede the development of pathogens (Heath, 1980). Thordal-Christensen (2003) proposed a minimum of four barriers that included (1) germination and penetration of the leaf epidermis by a pathogen, (2) the ability to overcome pre-formed physical and/or chemical barriers, (3) the ability to avoid the inducible defense responses that govern pre-penetration resistance, and (4) the ability to avoid detection by membrane bound and intracellular defense surveillance system. A microbe that can circumvent or suppress these four barriers, and establish a compatible interaction, is known as an adapted pathogen. Contrastingly, a microbe that is impeded by any of the mechanisms described above is unable to establish a compatible interaction and is declared a nonhost pathogen (Zimmerli et al., 2004).

The identification of overlap between host and nonhost resistance prompted the development of models that integrate membrane and intracellular signaling pathways involved in plant immunity (Thordal-Christensen, 2003; Schulze-Lefert and Panstruga, 2011). Schulze-Lefert and Panstruga (2011) proposed an evolutionary model wherein the relative contribution of pattern recognition receptors and nucleotide-binding, leucine-rich repeat proteins in conditioning resistance would be inversely correlated based on the phylogenetic distance to the host species. While an intriguing proposal, the majority of research on the molecular mechanisms underlying nonhost resistance has been derived from nonhost systems that are phylogenetically distant to the host system (Fan and Doerner, 2012; Gill et al., 2015a). Therefore, it will be necessary to identify biological systems that span the transition from host to nonhost. Bettgenhaeuser et al. (2014) defined the transition from host to nonhost with four states: host, intermediate host, intermediate nonhost, and nonhost. Classification into these four states depends on the degree of infection relative to a representative set of accessions from a species. In particular, intermediate classification will often involve a small number of accessions being colonized or allowing for the completion of a pathogen’s life cycle (Bettgenhaeuser et al., 2014). Investigating systems on the boundary requires the development of appropriate phenotypic assays, which are often distinct from those used in host systems.

Several microscopy-based approaches have been developed to interrogate host-nonhost pathosystems. Shafiei et al. (2007) found that early barriers conditioned nonhost resistance in *Arabidopsis thaliana* to *Puccinia triticina*. This was predominantly observed as a reduction in the ability for germ tubes to identify stoma and concomitant reduction in haustorial formation (Shafiei et al., 2007). Genetic dissection of guard cell death and substomatal vesicle formation found independent architectures, suggesting that several layers of microbial perception limit the development of *P. triticina* on *A. thaliana*. In contrast, resistance in *B. distachyon* to *P. graminis* f. sp. *tritici* manifested as a reduction in the formation of penetration pegs, substomatal vesicles, and primary hyphae, whereas appressoria formation was unaffected (Figueroa et al., 2013). Ayliffe et al. (2010, 2011, 2013) found a general requirement for microscopy-based approaches to visualize the development of infection structures in the interactions of *B. distachyon* and rice (*Oryza sativa*) with several cereal rusts, although some symptoms on *B. distachyon* were macroscopically visible. Microscopy was important in establishing a sequential reduction in oriented fungal growth, appressoria, and haustoria formation in the interaction of six monocot species, including *B. distachyon*, with the switchgrass rust *P. emaculata* (Gill et al., 2015b). In addition to early infection structures, the use of microscopy by Jafary et al. (2006, 2008) was critical for determining the number of pustules forming per unit area in the interaction of barley and *P. triticina*. In this instance the assay was essential, as the majority of the differential phenotypes between accessions were exhibited as variation in pustule formation rather than colonization.

In this report, we describe the interactions of barley and *B. distachyon* with *P. striiformis* f. sp. *tritici* as representative pathosystems for describing intermediate host and intermediate nonhost resistance, respectively. We take advantage of the stepwise infection process of *P. striiformis* that begins with intercellular colonization of leaves and then transitions to pustule formation (Hovmøller et al., 2011). We develop a complimentary pair of phenotypic assays, pCOL and pPUST, to estimate the colonization and pustule formation of *P. striiformis*, and apply them in the context of host, intermediate host, and intermediate nonhost systems to show that the frequency of infection decreases with evolutionary divergence from the host species. In parallel, we observe that the pathogen’s ability to complete its life cycle decreased faster than its ability to colonize leaf tissue with lower incidence of pustules observed in the intermediate nonhost system than in the intermediate host system.

## Materials and Methods

### Plant and Fungal Materials

Barley accessions were obtained from the United States Department of Agriculture Germplasm Resource Information Network (Aberdeen, ID, USA), the James Hutton Institute (Dundee, UK), Okayama University (Okayama, Japan), the Leibniz-Institut für Pflanzengenetik und Kulturpflanzen forschung (Gatersleben, Germany), the Estación Experimental de Aula Dei, Consejo Superior de Investigaciones Científicas (Madrid, Spain), Oregon State University (Corvallis, OR, USA), Washington State University (Pullman, WA, USA), and Wageningen University and Research Centre (Wageningen, Netherlands), (Supplementary Table S1). *B. distachyon* accessions were obtained from the John Innes Centre (Norwich, UK), Aberystwyth University (Aberystwyth, UK), (Draper et al., 2001; Brkljacic et al., 2011; Mur et al., 2011; Catalán et al., 2012), Sabancı University (Filiz et al., 2009), USDA-ARS (Garvin et al., 2008), and Universidad Politécnica de Madrid. The *Brachypodium* species complex has only been recently resolved (López-Alvarez et al., 2015) and our *B. distachyon* diversity set may also contain a few *B. hybridum* accessions (Supplementary Table S2). All plants underwent single seed descent before performing pathogen assays. *P. striiformis* f. sp. *tritici* isolates 08/21 and 08/501 were collected in the United Kingdom in 2008 and maintained at the National Institute of Agricultural Botany (NIAB) on a susceptible cultivar of wheat. *P. striiformis* f. sp. *hordei* isolate B01/2 was collected in the United Kingdom in 2001 and maintained at NIAB on the susceptible barley cultivar Cassata. *P. striiformis* urediniospores were stored at 6°C after collection.

### Pathogen Assays

Plants were sown in 1 L pots containing peat-based compost in groups of four, using eight seeds per accession. Plants were grown at 18°C day and 11°C night using a 16 h light and 8 h dark cycle in a controlled environment chamber at NIAB, with lighting provided by metal halide bulbs (Philips MASTER HPI-T Plus 400W/645 E40) with an average light intensity of 5.6 klux. Barley seedlings were inoculated at 14 days after sowing, where first leaves were fully expanded and the second leaf was just beginning to emerge. *B. distachyon* seedlings were inoculated 4 weeks after sowing at the four to five leaf stage (BBCH14/BBCH15; Onda et al., 2015). Urediniospores of *P. striiformis* were suspended in talcum powder, at a 1:16 ratio of urediniospores to talcum powder based on weight. Compressed air was used to inoculate seedlings on a spinning platform. After inoculation, seedlings were placed in a sealed bag and stored at 6°C for 48 h to increase humidity for successful germination of urediniospores. Subsequently, plants were returned to the growth chamber for the optimal development of *P. striiformis*.

### Macroscopic Phenotyping

Macroscopic symptoms were evaluated on the first leaf (barley) or fourth/fifth leaf (*B. distachyon*) of all seedlings at 14 days post-inoculation (dpi). For barley, the observation of chlorosis (yellowing) and *P. striiformis* infection (pustule formation) phenotypes were scored on a nine-point scale from 0 to 4, with increments of 0.5. For *B. distachyon*, the observation of browning (brown necrotic-like phenotype) was scored using the same scale. Chlorosis, browning, and the formation of pustules were scored on a scale from 0 to 4, with 0 given for asymptomatic leaves, i.e., no chlorosis, browning, or pustules, and a score of 4 indicated full expression of the respective phenotype (i.e., 100% of the surface area). For chlorosis or browning, a score of 4 would indicate the entire leaf was yellow or brown, respectively, whereas a score of 4 for pustule formation was given to leaves with pustules present on the entirety of the leaf. For all three assays, the resolution was based on a 1 cm^2^ grid. The McNeal scale is based on the observed disease symptoms: 0 (immune; no visible symptoms), 1 (necrotic/chlorotic flecks without sporulation), 2 [necrotic/chlorotic stripes (NCS) without sporulation], 3 (trace sporulation with NCS), 4 (light sporulation with NCS), 5 (intermediate sporulation with NCS), 6 (moderate sporulation with NCS), 7 (abundant sporulation with NCS), 8 (abundant sporulation with chlorosis), and 9 (abundant pustule formation, without chlorosis), (McNeal et al., 1971).

### Microscopic Phenotyping

We adapted a protocol described by Ayliffe et al. (2011, 2013) that uses wheat germ agglutinin (WGA; a lectin that interacts with chitin oligomers) conjugated with the fluorescein isothiocyanate (FITC) fluorophore to visualize the intercellular growth and pustule formation on infected leaves. Leaves were harvested at 14 dpi and placed in 15 mL centrifuge tubes containing 1.0 M KOH with a droplet of surfactant (Silwet L-77, Loveland Industries Ltd.). Leaves were cleared by incubating in the KOH solution at 37°C for 12 to 16 h. Subsequently, the KOH solution was decanted and leaves were neutralized by washing three times in 50 mM Tris at pH 7.5. After decanting of the final wash solution, a 1.0 mL stain solution (20 μg/mL WGA-FITC (L4895-10MG; Sigma–Aldrich) in 50 mM Tris at pH 7.5) was applied to the leaves. Leaf tissue was incubated overnight, then washed with water, mounted, and observed under blue light excitation on a fluorescence microscope with a GFP filter. We developed two microscopy-based phenotypic assays to estimate the percent of leaf colonized (pCOL) and percent of leaf harboring pustules (pPUST) of *P. striiformis.* The microscopic assays were developed to quickly evaluate disjoint fields of view (FOV) covering the surface area of the leaf by scanning a mounted leaf segment along either side of the longitudinal axis for barley or the longitudinal axis for *B. distachyon. P. striiformis* will often generate an initial infection site, which can include a substomatal vesicle, haustorial mother cell, and some additional development of intracellular hyphae in resistant interactions (Ayliffe et al., 2011; Hovmøller et al., 2011). Based on this observation, within each FOV an estimate is generated based on a convex hull outlined by *P. striiformis* hyphal growth to be less than 15%, between 15 and 50%, or greater than 50% of the FOV area and given scores of 0, 0.5, or 1, respectively. A convex hull is defined as the minimal area that contains the full extent of colonization of *P. striiformis* within a FOV. Disjoint convex hulls may occur within the same FOV and are used to collectively estimate the percent colonization in each FOV. Each score was recorded, with the final pCOL score determined by averaging these scores based on the total number of FOVs evaluated and ranged from 0 to 100%. pPUST was evaluated in a similar manner, although convex hulls were defined by the clustering pattern of *P. striiformis* pustules. Due to the difference in scale of barley and *B. distachyon* leaves, a 5x objective with a FOV of 2.72 mm × 2.04 mm and a 10x objective with FOV of 1.36 mm × 1.02 mm were used for barley and *B. distachyon*, respectively.

### Experimental Design and Statistical Analysis

For experiments with the interaction of barley and *P. striiformis* f. sp. *tritici* isolate 08/21, two sets of three leaves were evaluated to generate a single macroscopic and microscopic phenotypic value. For experiments with the interaction of barley and *P. striiformis* f. sp. *hordei* isolate B01/2, two sets of three leaves were evaluated to generate a single macroscopic phenotypic value and with the first set of three leaves used for microscopy. For experiments with the interaction of *B. distachyon* and *P. striiformis* f. sp. *tritici*, three leaves were evaluated to generate a single macroscopic and microscopic phenotypic value from a single experiment. This was repeated with *P. striiformis* f. sp. *tritici* isolates 08/21 and 08/501, which are highly related based on transcriptome sequencing (Hubbard et al., 2015). Data presented is from the first replicate of all experiments to demonstrate the robustness of the microscopic assay and to preserve the macroscopic-microscopic association. Pearson rank correlation coefficients (ρ) were determined using the *cor* command in R (v3.1.0).

## Results

**FIGURE 1:**
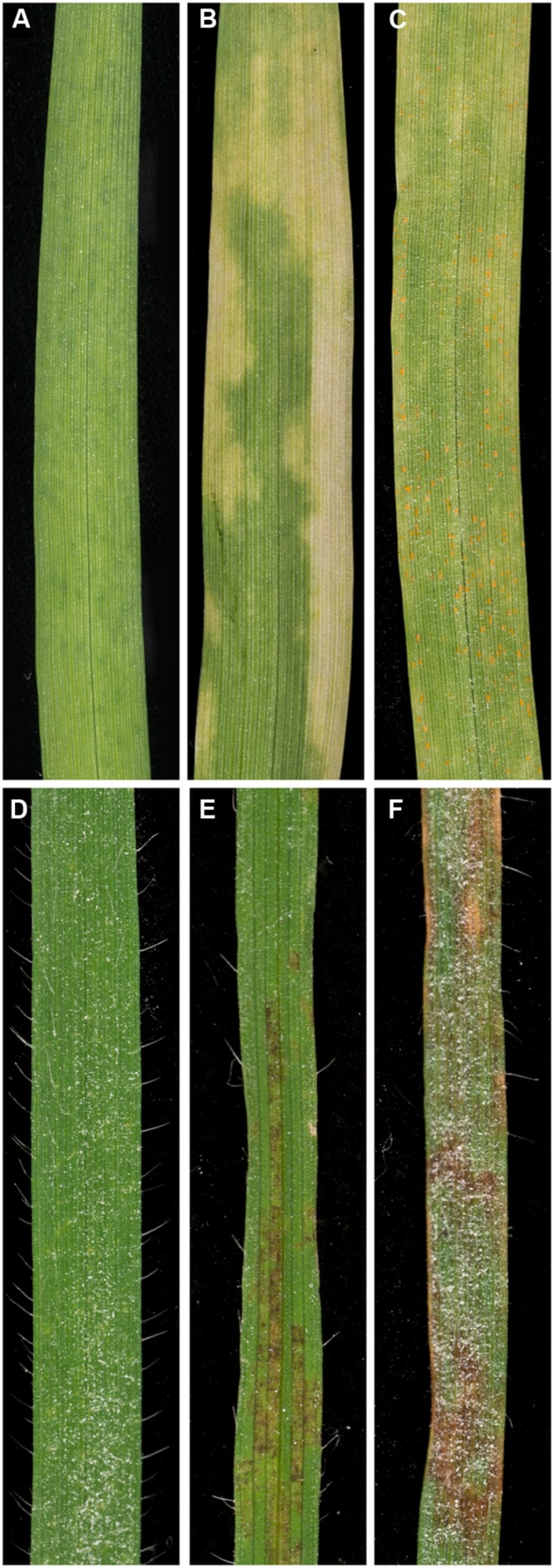
**Macroscopic phenotypes observed on barley and *Brachypodium distachyon* accessions challenged with *Puccinia striiformis* f. sp. *tritici*.** Barley accessions challenged with *P. striiformis* f. sp. *tritici* were generally categorized into three groups: **(A)** immune (no observable macroscopic symptoms; accession Abed Binder 12), **(B)** chlorotic (accessions displaying varying degrees of chlorosis; accession Foster), and **(C)** compatible (pustules observed, indicative of life cycle completion; accession Manchuria). *B. distachyon* accessions challenged with *P. striiformis* f. sp. *tritici* displayed either **(D)** immunity (accession ABR6), **(E)** moderate leaf browning (accession Bd21), or **(F)** severe leaf browning (accession Tek-4).

The interactions of *P. striiformis* f. sp. *tritici* with barley and *B. distachyon* have been proposed as intermediate host and intermediate nonhost systems, respectively (Bettgenhaeuser et al., 2014). Previous research has demonstrated the occurrence of susceptibility to this pathogen in both species (Straib, 1937; Draper et al., 2001), but the frequency of susceptibility has never been systematically studied in a large collection of germplasm. Our initial approach was to screen a large collection of barley and *B. distachyon* germplasm with *P. striiformis* f. sp. *tritici* to establish the frequency and degree of susceptibility. To do this, we inoculated and macroscopically phenotyped a panel of 237 barley and 210 *B. distachyon* accessions with *P. striiformis* f. sp. *tritici* isolate 08/21. In barley, the majority of accessions challenged with *P. striiformis* f. sp. *tritici* were immune, i.e., green and free of disease symptoms (78%; 184/237; **Figure 1A**). However, a significant proportion (22%; 54/237) of genotypes exhibited varying degrees of chlorosis (**Figure 1B**). Within this set of accessions exhibiting chlorosis, we observed a few instances (3%; 7/237) of the completion of pathogen life cycle, namely, pustule formation (**Figure 1C**). In contrast to barley, we did not observe the completion of pathogen life cycle in the interaction of *B. distachyon* and *P. striiformis* f. sp. *tritici*. While most accessions exhibited an immune phenotype (86%; 180/210; **Figure 1D**), another phenotype, in the form of leaf browning was observed (14%; 30/210; **Figures 1E,F**). It was unclear whether chlorosis or browning were direct responses to pathogen ingress or general stress responses. However, no chlorotic or browning symptoms were evident on leaves in the absence of *P. striiformis* f. sp. *tritici*. In addition, the patterning of chlorosis in barley and browning in *B. distachyon* was not random and was often associated with stripe-like patterns on the leaf. Therefore, we hypothesized that these two phenotypes were analogous between these two interactions and were associated specifically with *P. striiformis* f. sp. *tritici*.

**FIGURE 2:**
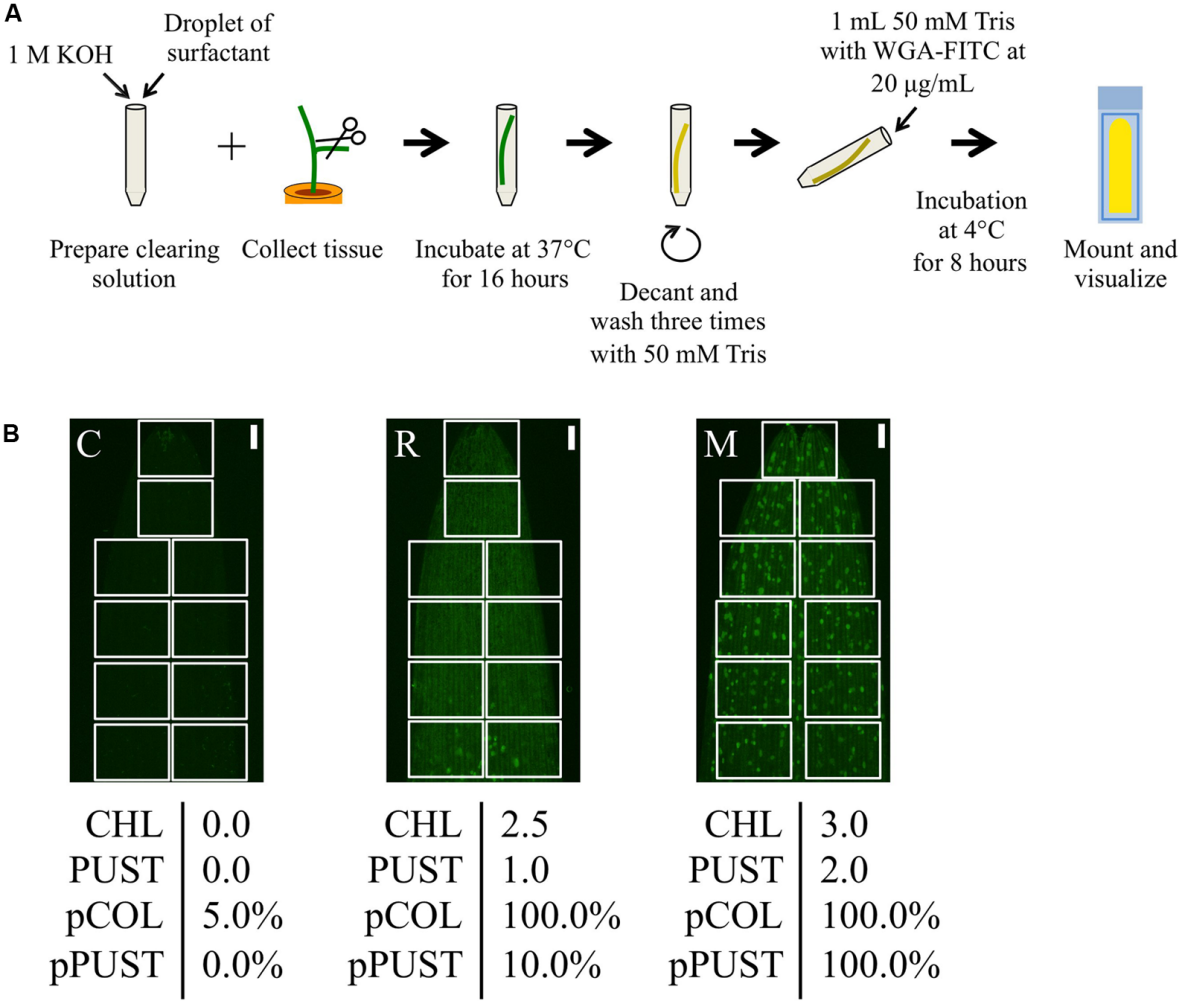
**Quantitative microscopic phenotyping of *P. striiformis* colonization and pustule formation using pCOL and pPUST on barley. (A)** A modified WGA-FITC staining protocol based on the procedure proposed by Ayliffe et al. (2011, 2013). **(B)** Representative samples with macroscopic phenotypes for chlorosis (CHL) and pustule formation (PUST) were microscopically analyzed using pCOL and pPUST based on stereo micrographs of the first leaf. The microscopic assays were developed for quick evaluation through evaluating disjoint fields of view (FOV; white boxes) covering the surface area of the leaf by scanning a mounted leaf segment along either side of the longitudinal axis for barley or centered on the longitudinal axis for *B. distachyon*. For pCOL, an estimate is generated within each FOV based on a convex hull defined by *P. striiformis* hyphal growth to be less than 15%, between 15 and 50%, or greater than 50% of the FOV area and given scores of 0, 0.5, or 1, respectively. A convex hull is defined as the minimal area that contains the full extent of colonization of *P. striiformis* within a FOV. Disjoint convex hulls may occur within the same FOV and are used to collectively estimate the percent colonization in each FOV. Each score was recorded, with the final score determined by averaging these scores based on the total number of FOVs evaluated and ranged from 0 to 100%. pPUST was evaluated in a similar manner, although convex hulls were defined by the clustering pattern of *P. striiformis* pustules. From left to right, accessions CIho 4196 (C), Russell (R), and Manchuria (M). The scale bar is 1 mm.

**FIGURE 3:**
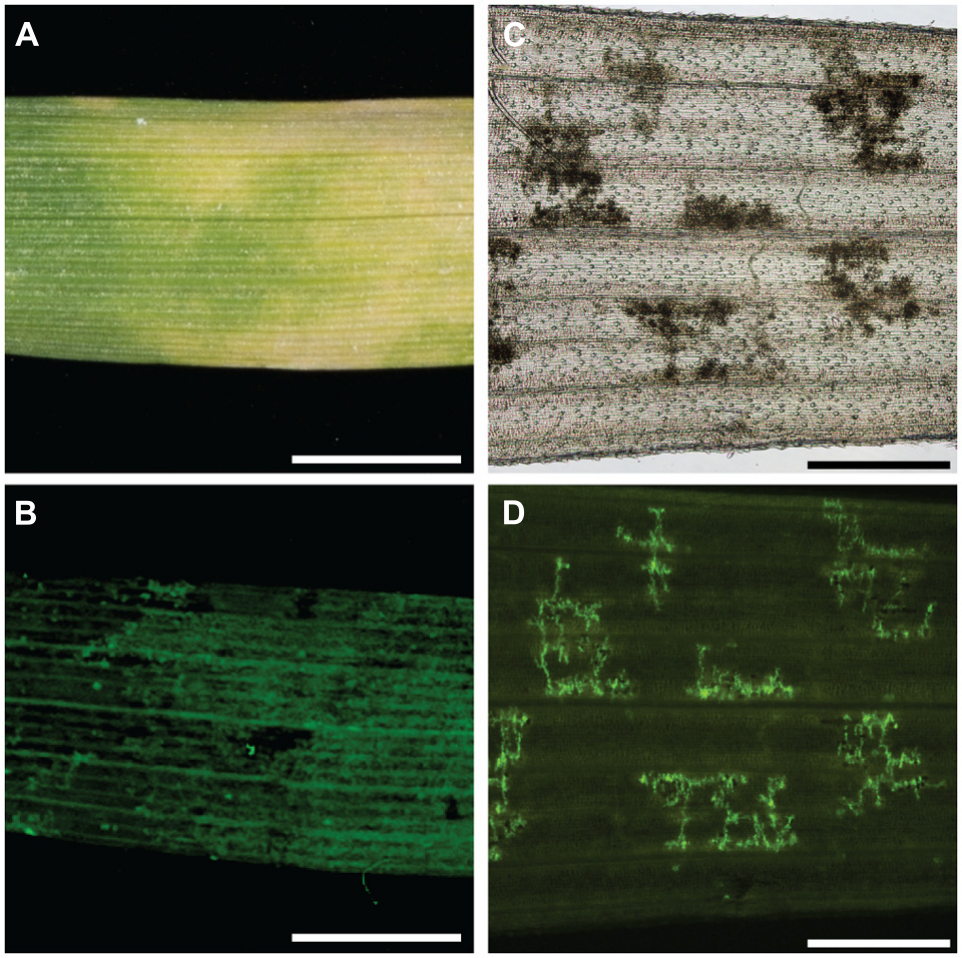
**Colocalization of chlorosis in barley and leaf browning in *B. distachyon* with *P. striiformis* f. sp. *tritici* hyphae. (A)** Macroscopic chlorosis in barley accession Steptoe challenged with *P. striiformis* f. sp. *tritici* isolate 08/21. **(B)** Fluorescent micrograph of **(A)** stained with WGA-FITC. **(C)** Micrograph of a cleared leaf of *B. distachyon* accession Bd21 leaf exhibiting the leaf browning phenotype. **(D)** Fluorescent micrograph of **(C)** stained with WGA-FITC. The scale bar is 5 mm for **(A,B)** and 500 μm for **(C,D)**.

**FIGURE 4:**
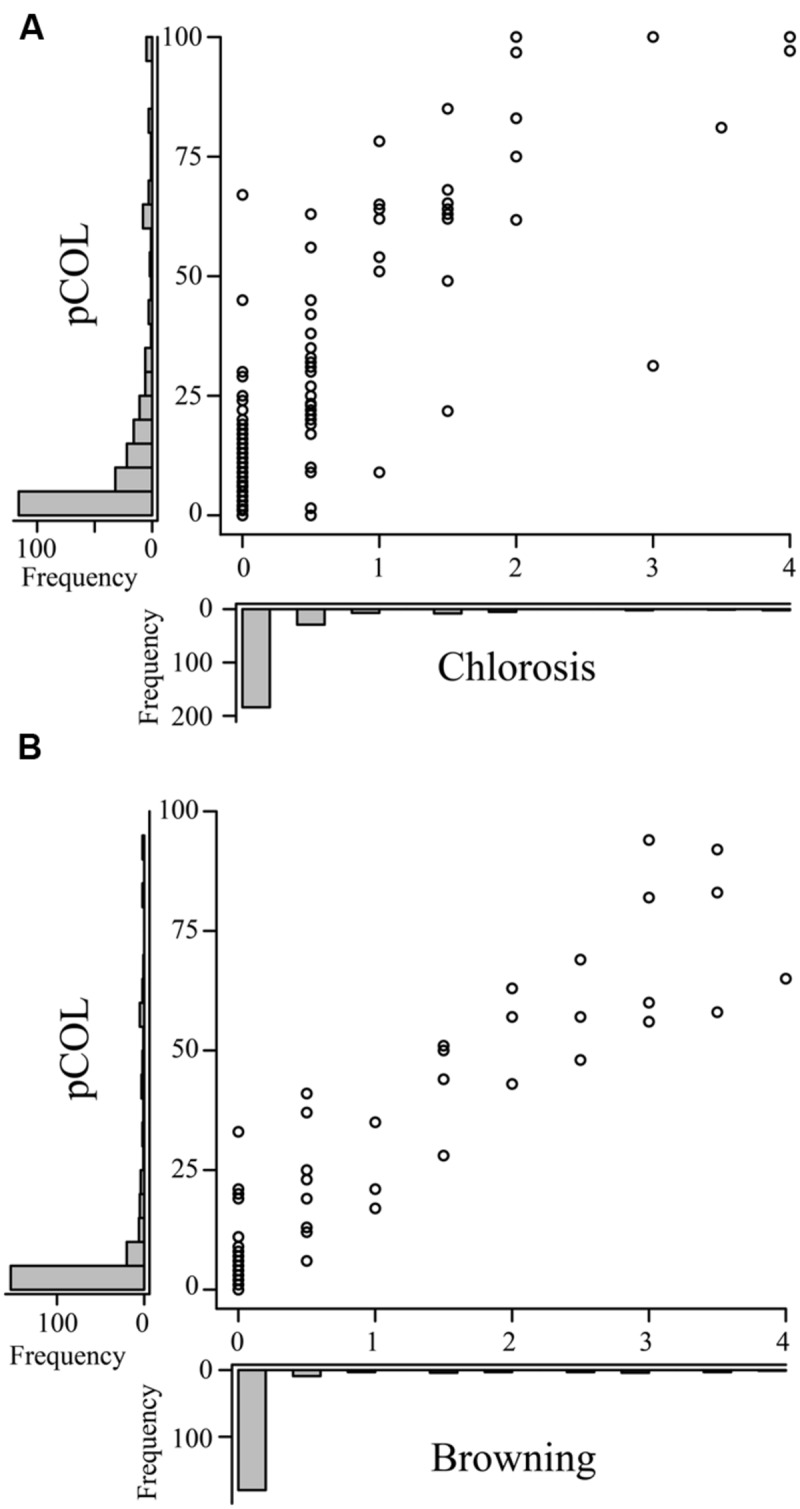
**Macroscopic symptoms of chlorosis and leaf browning in barley and *B. distachyon* are associated with leaf colonization by *P. striiformis* f. sp. *tritici*.** Plots comparing the correlation of macroscopic chlorosis **(A)** and leaf browning **(B)**, (*x*-axes) with microscopic pCOL phenotypes (*y*-axes) in barley and *B. distachyon*, respectively, challenged with *P. striiformis* f. sp. *tritici* isolate 08/21. Histograms showing the frequency of phenotypic observations are displayed on the corresponding axis for the data shown in the plot.

To investigate whether chlorosis and browning were a direct result of *P. striiformis* f. sp. *tritici* colonization, we adapted a staining method in combination with fluorescence microscopy to visualize the presence of hyphal structures (**Figure 2A**; Ayliffe et al., 2011, 2013). Initial observations demonstrated little or no hyphal growth in immune barley and *B. distachyon* accessions. In contrast, barley and *B. distachyon* accessions harboring chlorosis and browning phenotypes appeared to have substantial *P. striiformis* f. sp. *tritici* hyphae (**Figure 3**). To quantify the association of these two phenotypes with *P. striiformis* f. sp. *tritici* infection, it was necessary to develop a phenotypic assay to quantify the area of the leaf infected by *P. striiformis* f. sp. *tritici* (pCOL; **Figure 2B**). When we applied pCOL to barley, a strong association was observed between accessions displaying chlorotic symptoms and colonization of *P. striiformis* f. sp. *tritici* (ρ = 0.84; **Figure 4A**). A few exceptions did exist, including a few accessions displaying chlorotic symptoms but comparatively reduced pCOL (**Figure 4A**). While chlorosis does not fully predict pCOL, the correlation suggests that chlorosis in barley was likely a response to colonization by *P. striiformis* f. sp. *tritici*. In *B. distachyon*, the association between browning and colonization of *P. striiformis* f. sp. *tritici* was robust (ρ = 0.93; **Figure 4B**). This association is tightly regulated, as the coupling of these two phenotypes was observed down to the resolution of hyphae at the microscopic level (**Figure 3**). A similar association was observed after testing a subset of accessions with *P. striiformis* f. sp. *tritici* isolate 08/501. Taken together, these results demonstrate that chlorosis and leaf browning are associated specifically with colonization by *P. striiformis* f. sp. *tritici* in barley and *B. distachyon*, respectively.

**FIGURE 5:**
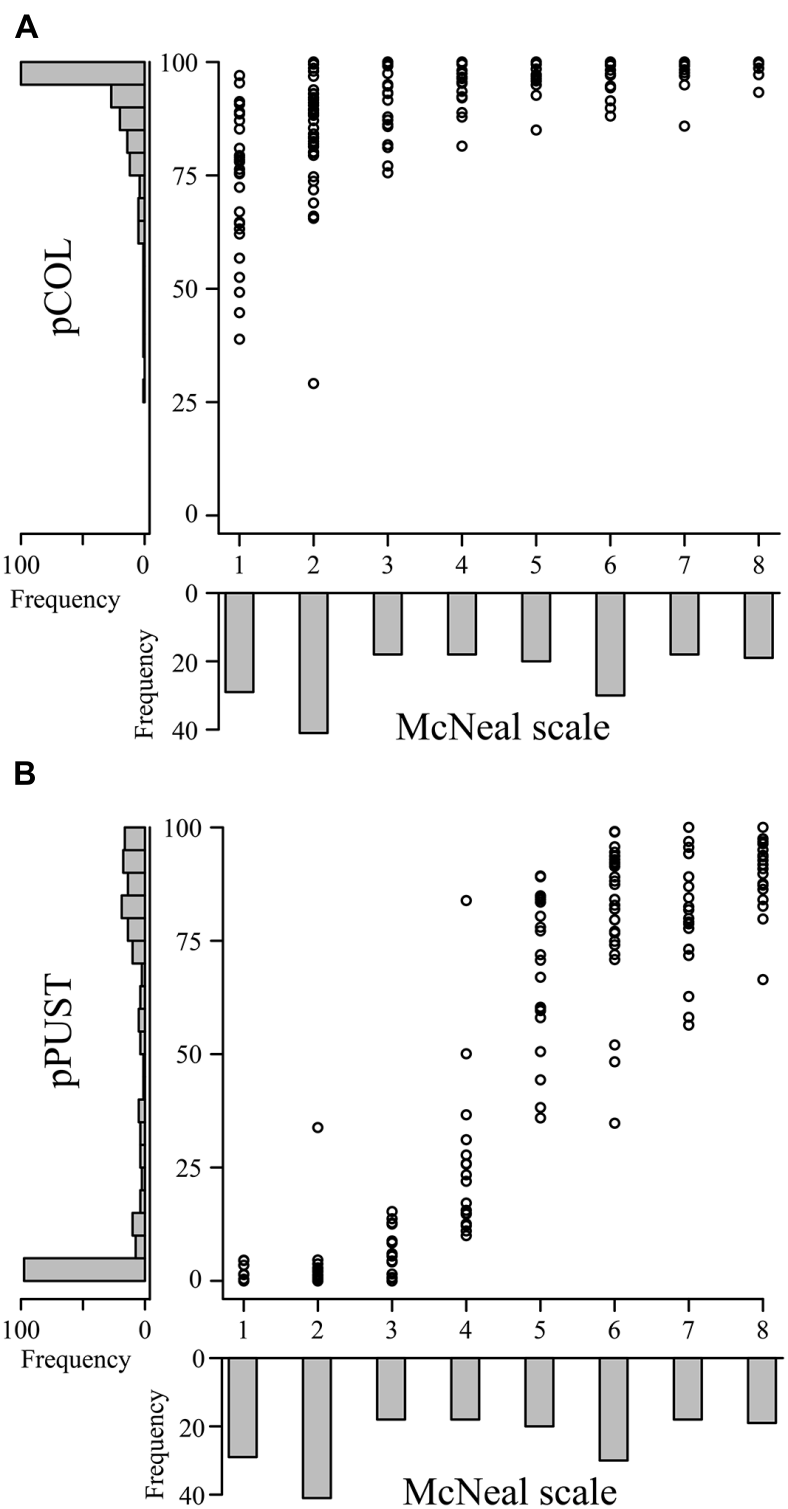
**Comparison of macroscopic and microscopic phenotypes in the barley-*P. striiformis* f. sp. *hordei* interaction.** Plots comparing the macroscopic McNeal scale (*x*-axes) with the microscopic phenotypes pCOL **(A)** and pPUST **(B)**, (*y*-axes) in barley accessions challenged with *P. striiformis* f. sp. *hordei* isolate B01/2. Histograms showing the frequency of phenotypic observations are displayed on the corresponding axis for the data shown in the plot.

To provide context for the differentiation between host, intermediate host, intermediate nonhost, and nonhost systems, it is essential to characterize each system in detail with identical phenotypic assays. On this premise, we assessed the applicability of pCOL on the barley–barley stripe rust (*P. striiformis* f. sp. *hordei*) system. To do this, we inoculated a collection of barley accessions with *P. striiformis* f. sp. *hordei* isolate B01/2 and phenotyped using the ten-point scale proposed by McNeal et al. (1971) and pCOL (**Figure 5A**). All accessions exhibited some degree of colonization with the lowest observed pCOL at 29.1% and only four accessions exhibiting less than 50% pCOL. The majority of accessions (90%; 173/193) displayed greater than 75% pCOL. When phenotyped using the McNeal scale, accessions exhibited phenotypes ranging from 1 (necrotic/chlorotic flecks without pustule formation) to 8 (abundant pustule formation with chlorosis) in their phenotype and had a relatively equal distribution across the McNeal scale (**Figure 5**). Strikingly, the majority of accessions (64%; 123/193; McNeal scale ≥3) showed some degree of pustule formation. This observation highlights the differentiation between host and nonhost systems and the need to develop a microscopic assay to specifically quantify pustule formation. Thus, we established a phenotypic assay to determine the percent of the leaf harboring pustules (pPUST; **Figure 2B**).

We observed a strong correlation between the McNeal and pPUST phenotypes (ρ = 0.92; **Figure 5B**). This association was expected, as the McNeal scale was developed for use on host systems, particularly for assessing the extent of pustule formation on infected leaves. However, a non-linear relationship was clearly evident between the McNeal and pPUST phenotypes. This suggested that the McNeal scale was not optimal for describing variation in leaf area with pustules, particularly when pustule density was between 25 and 75% of the leaf surface area. At these pustule densities pPUST is a more suitable phenotype as it identifies variation in a linear scale. Similarly, pCOL also captured additional variation in disease severity at the lower end of the McNeal scale (0 and 3). However, the McNeal scale may be describing additional variation as compared to pPUST when pustule density increased above 75% of the leaf surface area (scores of 7 or above). The McNeal scale takes into account greater pustule density and stress responses (necrosis/chlorosis) at scores greater than or equal to 7, which is not taken into account with the pPUST microscopic phenotype. Taken together, these differing phenotypic scales uncover additional variation in the interaction of barley-*P. striiformis* f. sp. *hordei* and can be used in conjunction with existing macroscopic phenotypic assays to provide greater resolution of the phenotypic variation observed within a large collection of accessions.

## Discussion

Pathosystems that exist in the transitory state between host and nonhost have been described as intermediate systems. Intermediate systems are proposed to involve a limited number of accessions being susceptible to a pathogen species, or limited numbers of isolates being able to infect a given plant species (Bettgenhaeuser et al., 2014). Few intermediate systems have been studied and we have a restricted understanding of the genetic architecture of resistance underlying such systems. This may be attributed to (1) a lack of robust, quantitative phenotypic assays applicable to these systems or (2) the inability to identify rare accessions that support colonization or the full life cycle of a pathogen. In this report, we have developed two quantitative microscopic assays and applied them to Poaceae–*P. striiformis* interactions to identify an intermediate host and intermediate nonhost system.

Dissection of the architecture of resistance in intermediate systems requires robust phenotypes. Robustness is a broad term that describes the favorable combination of resolution, accuracy, precision, throughput, and biological context of the phenotypic assay. These five criteria for assessment will be influenced by the nature of the information that can be assessed in any given pathosystem. Therefore, phenotypic assays require calibration to the system being studied and will differ depending upon the infection stage that is being observed (e.g., spore differentiation, haustoria formation, colonization, or pustule formation). For example, Jafary et al. (2006, 2008) used macroscopic observation of pustule formation to show the incidence of quantitative trait loci that govern intermediate host status in barley-*Puccinia* pathosystems. Similarly, macroscopic observation of life cycle completion has been used to identify gene-for-gene interactions in Poaceae–*B. graminis* and Poaceae–*M. oryzae* nonhost pathosystems (Tosa et al., 1987, 1988; Tosa and Sakai, 1990; Inukai et al., 2006; Nga et al., 2012). However, the phenotypes used in these systems are largely dependent upon the completion of the pathogens’ life cycle, something that may not always manifest in intermediate systems. Therefore, in intermediate systems, it is necessary to use microscopic phenotypes to assess the extent to which a pathogen infects a potential host. Indeed, microscopic evaluation has been used to demonstrate variation in spore differentiation and infection structure development of *Puccinia* rust fungi on *B. distachyon* (Figueroa et al., 2013). Similarly, Ayliffe et al. (2011) used a WGA-Alexa488 microscopy-based assay to successfully demonstrate that several cereal rusts including *P. graminis* f. sp. *tritici* were able to establish basic compatibility on rice. We adapted this protocol to develop two assays, pCOL and pPUST, for quantifying levels of colonization and pustule formation, respectively. Application of these assays to two intermediate Poaceae-stripe rust pathosystems allowed us to visualize the progression of this spreading pathogen. This revealed that resistance was conditioned at two different stages: colonization and life cycle completion (i.e., pustule formation).

The development of microscopic assays established a link between macroscopic observations of chlorosis and browning with the ingress of *P. striiformis* f. sp. *tritici* in challenged barley and *B. distachyon* leaves. Chlorosis and leaf browning are common phenotypic responses observed during plant–pathogen interactions (Mishra et al., 2015). However, they are also prototypical phenotypes implicated in a plethora of abiotic stress responses (Drew and Sisworo, 1977; Sicher, 1997; Shaibur et al., 2008). As such, ambiguity can exist as to the exact underlying cause of such phenotypes and this may impede their use in classical genetic analyses. This study has provided strong evidence that the macroscopic observations of chlorosis and leaf browning in barley and *B. distachyon*, respectively, are linked to leaf colonization by *P. striiformis* f. sp. *tritici*. Disambiguation of this response from other potential abiotic responses opens up the possibility of using this phenotype to dissect the genetic architecture of resistance to nonhost pathogens in intermediate systems in parallel with macroscopic phenotypic assays.

Conceptualization of nonhost resistance has moved away from binary approaches to continuous models. The terms intermediate host and intermediate nonhost have been introduced to describe systems that are in the transition between host and nonhost states (Bettgenhaeuser et al., 2014). Classification into these four states is dependent upon the analysis of representative sets of accessions from different plant species relative to host and nonhost pathogens. We applied pCOL and pPUST to representative samples of the barley-*P. striiformis* f. sp. *hordei* (host system), barley-*P. striiformis* f. sp. *tritici* (intermediate host system) and the *B. distachyon*-*P. striiformis* f. sp. *tritici* (intermediate nonhost system) pathosystems. These systems represent a stepwise progression from host through to intermediate nonhost status and clearly demonstrated a general reduction in infection with increasing evolutionary distance from the host. The incidence of life cycle completion decayed quicker than incidence of pustule formation as seen by the absence of pustules in the intermediate systems and limited variation for colonization in the host system. To further validate this hypothesis, it will be necessary to survey more pathosystems that span the evolutionary continuum between host and nonhost.

As a whole, this study has demonstrated the efficacy of the interaction of stripe rust with barley and *B. distachyon* as model systems for studying resistance to nonhost pathogens in intermediate systems. The development of two robust, quantitative phenotypic assays facilitated the disambiguation of asymptomatic phenotypes observed in nonhost interactions. Using these systems we now hope to dissect the genetic architecture underlying resistance.

## Conflict of Interest Statement

The authors declare that the research was conducted in the absence of any commercial or financial relationships that could be construed as a potential conflict of interest.
